# Supplementary material for: Solvent-non-solvent rapid-injection for preparing nanostructured materials from micelles to hydrogels
Source: Nat Commun. 2019 Aug 26;10:3855. doi: 10.1038/s41467-019-11804-7 (PMC6710291; doi:10.1038/s41467-019-11804-7)
Supplement: Supplementary file 1 — Supplementary Information [file 41467_2019_11804_MOESM1_ESM.pdf]

[Supporting Information]

Solvent-non-solvent rapid-injection for preparing nanostructured materials from micelles to hydrogels

Lang et al.

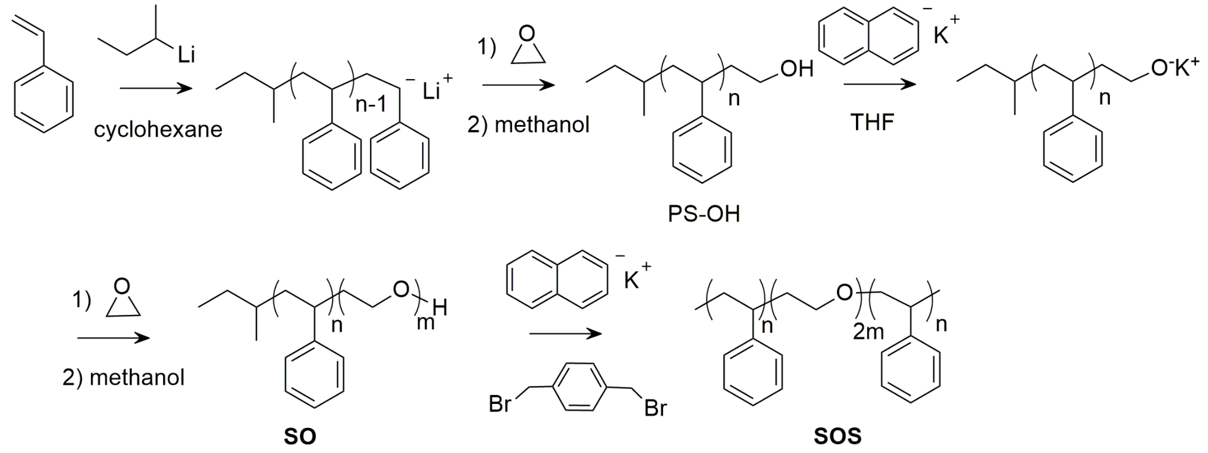

Supplementary Figure 1. Diblock copolymers SO, IO and BO were synthesized by using sequential anionic polymerization. Triblock copolymers SOS, IOI and BOB were then obtained by coupling the hydroxyl groups at the PEO end with α,α'-dibromo-*p*-xylene. The detailed synthetic route of SOS is shown below as an example.

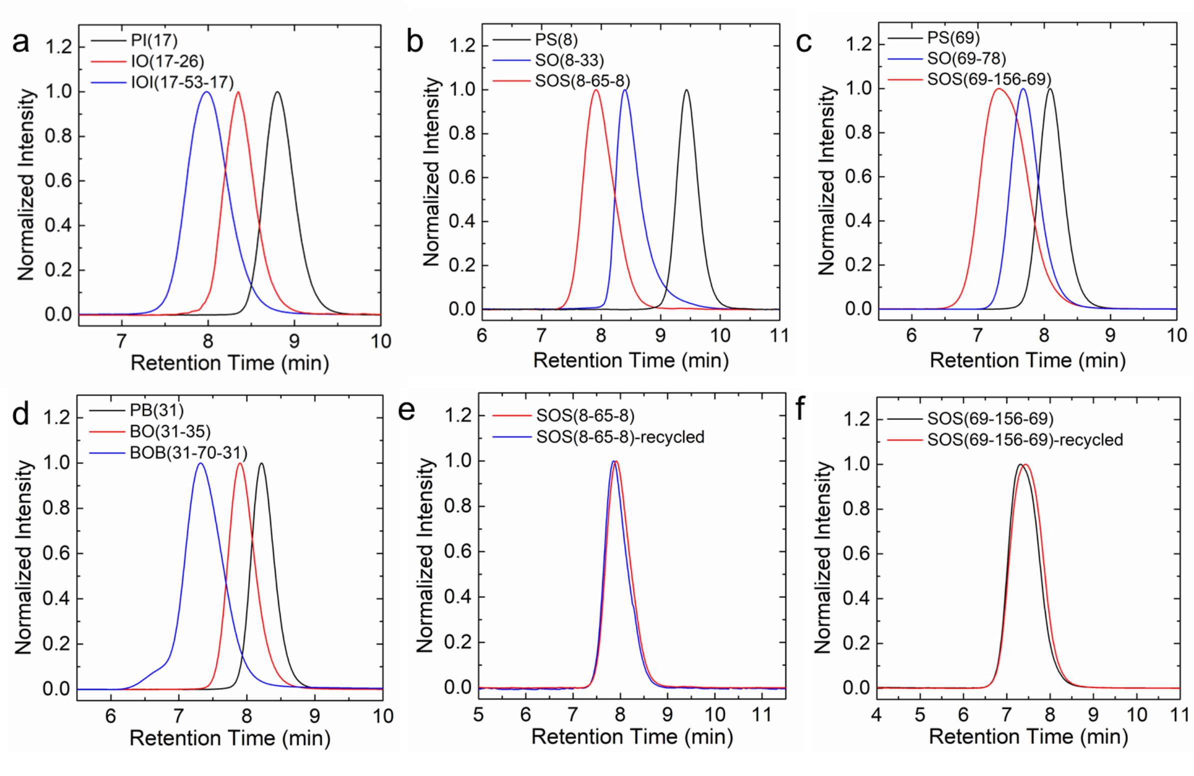

Supplementary Figure 2. Size-exclusion chromatography (SEC) traces of the homopolymers, the diblock copolymers, and the triblock copolymers used in the work. (a-d) Characterization of homopolymers, diblock copolymers, and triblock copolymers synthesized by sequential anionic polymerization. (e, f) Triblock copolymers can be recycled after forming hydrogels by dissolving in THF and precipitate in a 3:1 isopropanol/hexane solvent mixture.

Supplementary Table 1. Molecular weights, dispersity values, and poly(ethylene oxide) volume ratios for the diblock and triblock copolymers used in this study.

**Polymer**[a]	**$M_{n,total}$**[b] **(kg mol^{-1})**	**$M_{n,O}$**[c] **(kg mol^{-1})**	**$M_{n,hydrophobic}$**[c] **(kg mol^{-1})**	**f_O**[c]	**$Đ$**[d]
SO(8-33)	40.1	32.6	7.5	0.80	1.03
SOS(8-65-8)	68.8	56.0	12.8	0.80	1.03
SO(69-78)	146.9	77.9	69.0	0.51	1.03
SOS(69-156-69)	222.9	118.1	104.8	0.51	1.04
IO(17-26)	43.6	26.3	17.3	0.55	1.04
IOI(17-53-17)	54.5	32.9	21.7	0.55	1.05
BO(31-35)	66.1	35.1	31.0	0.46	1.03
BOB(31-70-31)	120.3	63.9	56.4	0.46	1.05

[a]In the paper, poly(isoprene), poly(styrene), poly(butadiene), and poly(ethylene oxide) are represented as I, S, B, and O, respectively. [b]Total number-average molecular weights of block copolymers were determined using SEC. Because the coupling efficiency is not 100%, there is diblock copolymer residual in the triblock copolymer sample. The $M_{n,total}$ of triblock copolymer measured from SEC is thus smaller than theoretical value (twice of the precursor diblock copolymer $M_{n,total}$). For homopolymer, previously reported *dn/dc* values were used[1]. For copolymers, the *dn/dc* values were calculated using ^{1}H NMR to get weighted average of *dn/dc* for different blocks. [c]M_n values of different blocks were calculated from $M_{n,total}$ and mass fraction of different blocks obtained from ^{1}H NMR. The O volume fraction (f_O) of the block copolymer was calculated using ^{1}H NMR data. The density values were obtained from Sigma-Aldrich, where the density of I is 0.906 g mL^{-1}, S is 1.04 g mL^{-1}, B is 0.86 g mL^{-1}, and O is 1.13 g mL^{-1} at 25 °C. [d]Dispersity index ($Đ = M_w/M_n$) was determined from SEC.

To quantitatively describe the solvent quality, interaction parameter χ between the polymers and solvents used in the study was listed in the table below. It has been established

that when the interaction parameter is smaller than 0.5, the interaction between the solvent and polymer is favorable (good solvent).[2,3] Whereas, when the interaction parameter is greater than 1, the interaction between the solvent and polymer is unfavorable (poor solvent).[2,3] As indicated in Supplementary Table 2, THF is a good solvent for S, I, B and O, while water is only a good solvent for O. Water is a poor solvent for S, I and B.

Supplementary Table 2. Polymer-solvent interaction parameter (25°C).

**Polymer**	$\chi_{\mathbf{THF}}$	$\chi_{\mathbf{water}}$
S	0.02[a]	6.26[a]
I	0.12[a]	7.10[a]
B	0.18[a]	6.93[a]
O	0.02[a]	0.45[b]

[a]Values calculated using solubility parameter of the solvents and homopolymers using Supplementary Equation 1,

$$\chi = \frac{V_s}{RT}\left(\delta_p - \delta_s\right)^2 \tag{1}$$

where χ is Flory interaction parameter, V_s is molar volume of the solvent, δ_p and δ_s are the solubility parameters of polymer and solvent respectively. [b]Previously reported value.[4]

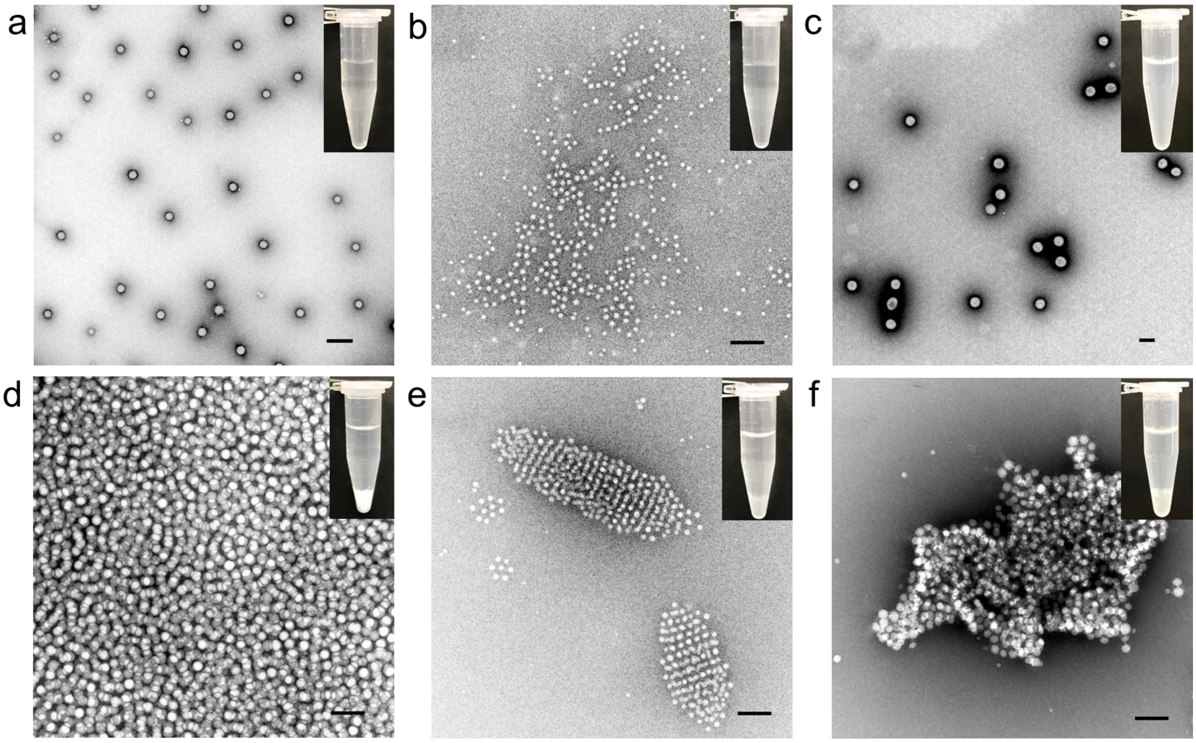

Supplementary Figure 3. TEM images of micelles and microgels formed via rapid-injection. Isolated micelles form in the dilute regime from (a) 0.05 wt% SOS(69-156-69) (b) 0.2 wt% IOI(17-53-17) (c) 0.05 wt% BOB(31-70-31) polymer solution in THF. Microgels formed in the semi-dilute regime from (d) 0.5 wt% SOS(69-156-69) (e) 1.5 wt% IOI(17-53-17) (f) 0.5 wt% BOB(31-70-31) polymer solution in THF. Scale bar: 200 nm.

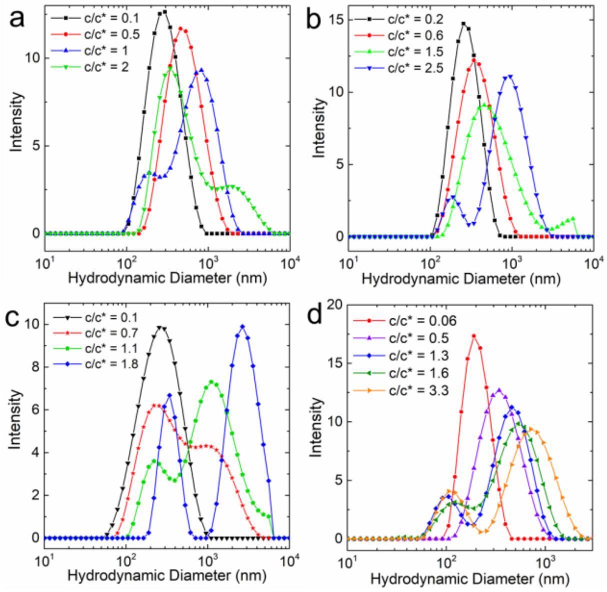

Supplementary Figure 4. DLS results for the colloidal micelles and microgels. Samples were formed when (a) SOS(69-156-69), (b) IOI(17-53-17), (c) BOB(31-70-31) and (d) SOS(8-65-

8) polymer solutions at dilute and semi-dilute regimes are injected into water. For microgel samples, the solution was allowed to settle, and the supernatant was used for the measurement. As concentration increases, the main peak of the sample shifts to larger size while the discrete micelle diameter stays relatively constant.

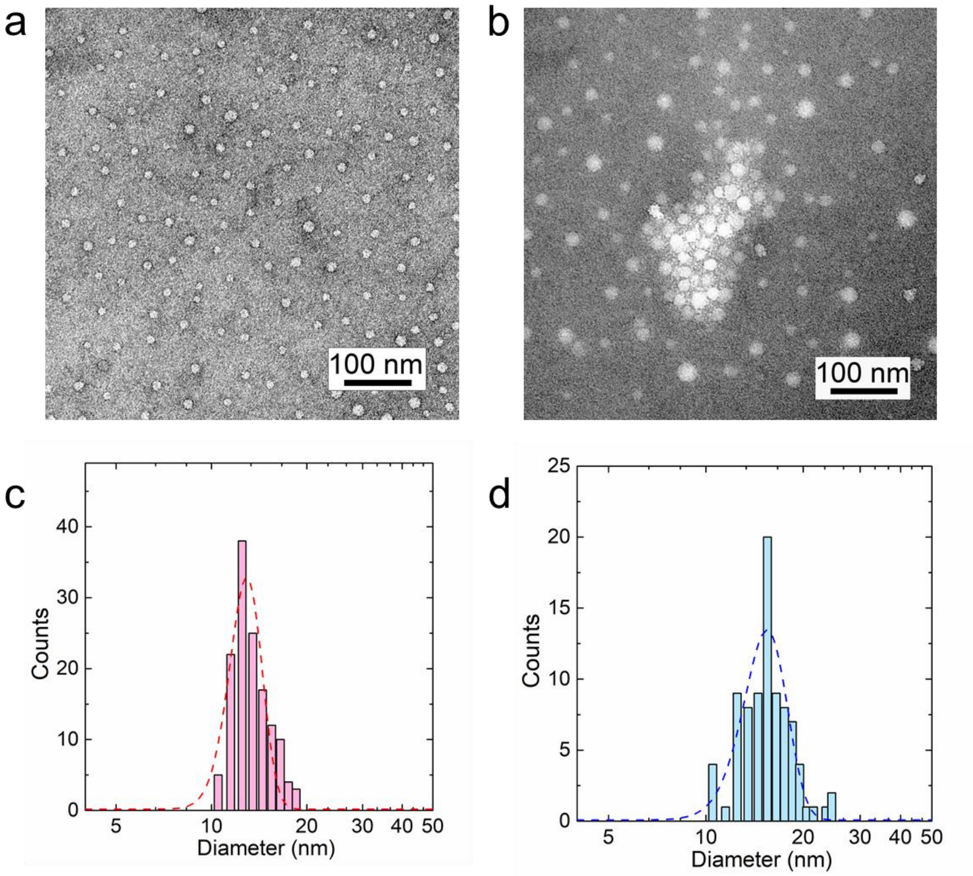

Supplementary Figure 5. Size analysis of discrete micelles from TEM characterization of SOS(8-65-8) sample. (a, c) 0.02 wt% SOS(8-65-8), c/c* = 0.025 (b, d) 1.0 wt% SOS(8-65-8), c/c* = 1.25. The diameter of the micelles in the 0.025 SOS(8-65-8) sample is 13.5±1.8 nm, which is similar to the diameter (15.8±2.7 nm) of the discrete micelles in 1.25 SOS(8-65-8) sample (c/c*) ("micelle/microgel mixture").

The aggregation number of the micelles can be estimated[5] from Supplementary Equation 2 using the micelle core size, molecular weight, and density of the hydrophobic block:

$$N_{\mathrm{agg}} = \frac{4\pi R^3 \rho N_{\mathrm{A}}}{3M} \qquad (2)$$

Where R is diameter of the micelle core, N_A is Avogadro's number, ρ and M are the density and the molecular weight of the hydrophobic block, respectively.

Supplementary Table 3. Micelle aggregation number estimated from micelle core radius R using TEM images of the microgels.

Sample	$M_{n,hydrophpobic}$ (kg mol^{-1})	R (nm)	N_{agg}
SOS(8-65-8)	12.8	9.7	184
SOS(69-156-69)	104.8	29.7	652
IOI(17-53-17)	21.7	14.6	791
BOB(31-70-31)	56.4	27.4	324

Supplementary Note 1. To calculate the theoretical overlap concentrations c^* of the polymers using Equation (1) in the main text, the polymer chain globule in the solution is approximated as a sphere with a radius of R_g (radius of gyration). The pervaded volume, V, is then calculated using Supplementary Equation 3.

$$V = \frac{4}{3}\pi {R_g}^3 \quad (3)$$

Number-average molecular weight (M_n) and radius of gyration (R_g) are obtained from the Wyatt multi-angle light scattering detector attached to the SEC.

Supplementary Table 4. Molecular weights, calculated overlap concentrations, experimentally determined overlap concentrations, and entanglement concentrations.

Sample	$M_{n,total}$ (kg mol^{-1})	c^*_{cal} (wt%)	c^*_{exp} (wt%)	$c_{e,exp}$ (wt%)
SOS(8-65-8)	68.8	1.0	0.8	5.0
SOS(69-156-69)	222.9	0.6	0.5	3.3
IOI(17-53-17)	54.5	1.4	1.0	6.0
BOB(31-70-31)	120.3	0.7	0.45	5.5

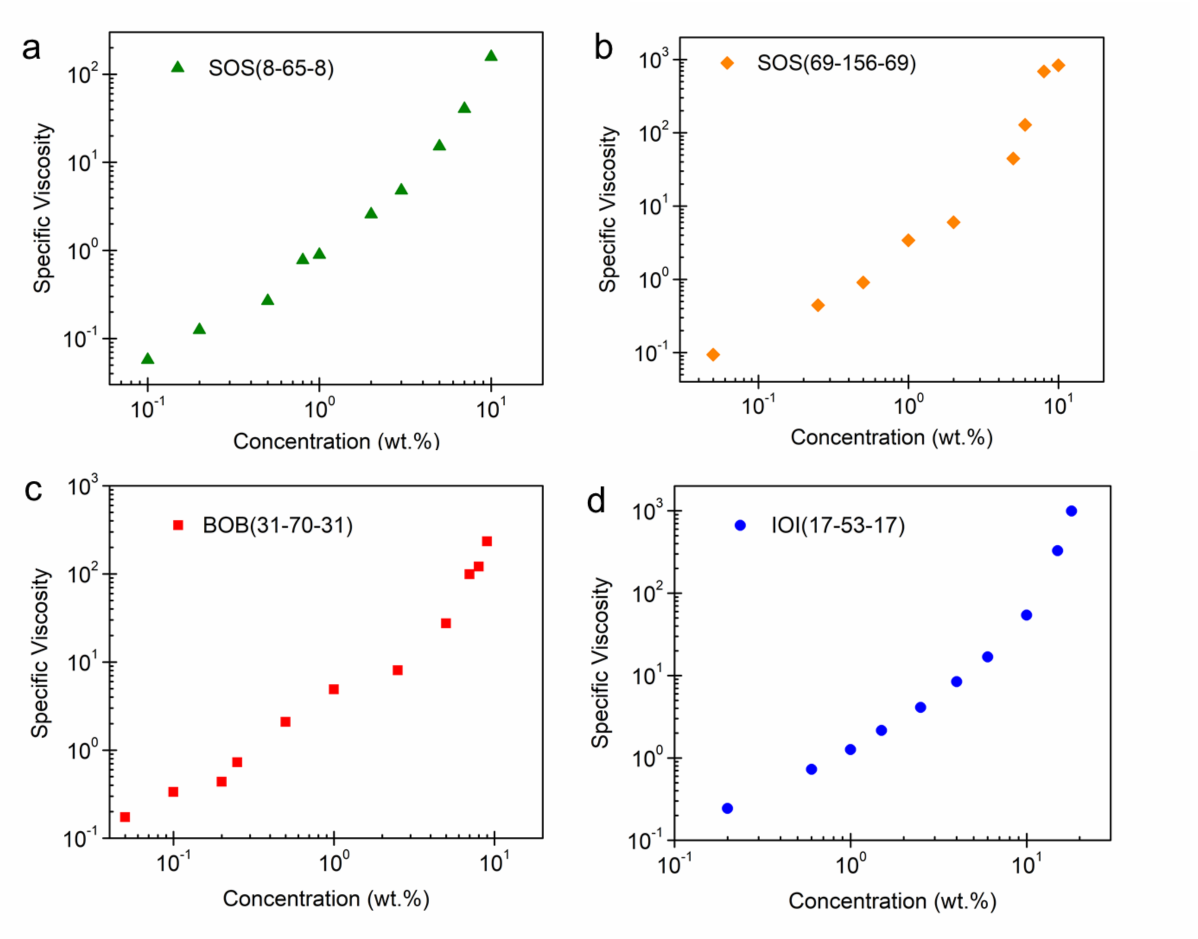

Supplementary Figure 6. Specific viscosity versus polymer concentration for the triblock copolymers used in the study. (a) SOS(8-65-8), (b) SOS(69-156-69) (c) BOB(31-70-31), and (d) IOI(17-53-17). The polymers were dissolved in THF. The scaling values on the plot indicate dilute, semi-dilute and concentrated regimes.

Supplementary Table 5. Water fraction of the microgels and hydrogels prepared using rapid injection.

Polymer	water fraction of hydrogel (wt%)	water fraction of microgel (wt%)
SOS(8-65-8)	94.8	81.9
SOS(69-156-69)	93.1	83.4
BOB(31-70-31)	92.8	77.5
IOI(17-53-17)	93.7	74.6

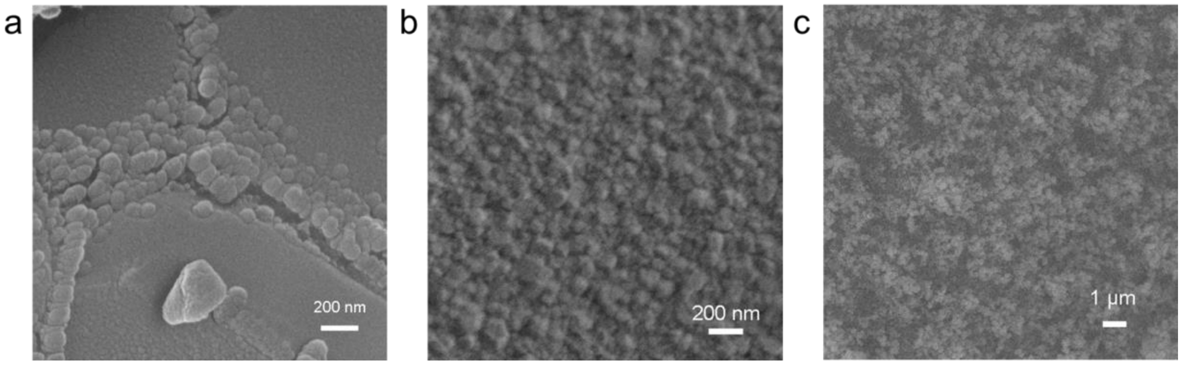

Supplementary Figure 7. Cryo-SEM images of the internal structure of the hydrogel using rapid-injection. Hydrogels formed using a (a) 10 wt% SOS(69-156-69) THF solution, (b) 10 wt% BOB(31-70-31) THF solution, and (c) 10 wt% IOI(17-53-17) THF solution. All three images indicate that the cellular network wall for the hydrogel consists of self-assembled micelles.

The scattering profile of the hydrogels was analyzed by considering the dependence of the scattering signal of the number of scatterers in the system, *n*, and the scattering contrast in the system, which is manifested from the hydrophobic micelle core vs water/PEO medium. Supplementary Table 6 shows calculated values for the scattering length density differences ($\Delta\rho$) between the hydrophobic micelle cores (S, I, and B) and the hydrophilic matrix (O and water). For the SOS samples, the scattering contrast is minimal, and meaningful data was not obtainable, especially using a lab source SAXS instrument.

For microgel samples, the decrease in the scattering signal is a result of a smaller scattering volume (the microgel solution is loaded in a 1.5 mm thick quartz capillary where the thickness of the hydrogel is ~5 mm), which leads to an overall reduction in the number of scatterers (hydrophobic micelle cores) (Supplementary Figure 8).

Supplementary Table 6. Calculated scattering length density values ρ for different hydrophobic blocks and scattering length density differences Δρ compared with O and water respectively. ρ_{PEO} is 10.49×10^{10} cm^{-2} and ρ_{water} is 9.441×10^{10} cm^{-2}.

Polymer	ρ (10^{10} cm^{-2})	$\lvert\rho-\rho_{PEO}\rvert$ (10^{10} cm^{-2})	$\lvert\rho-\rho_{water}\rvert$ (10^{10} cm^{-2})
S	9.516	0.974	0.075
I	8.599	1.891	0.842
B	8.115	2.375	1.326

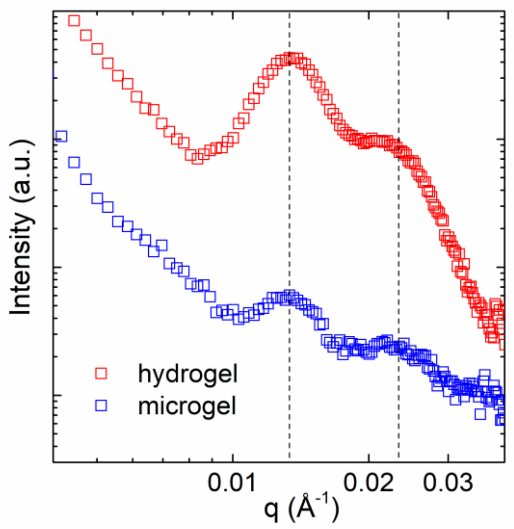

Supplementary Figure 8. SAXS scattering patterns for microgel and hydrogel samples from rapid-injection of 2.5 wt% and 15 wt% IOI(17-53-17) THF solutions. Both samples exhibit primary scattering peaks at identical *q* values arising from the micelle structure factor, indicating that both the micelle core center-to-center distances in the microgel and hydrogel are essentially the same. Different scattering intensities of IOI microgel and hydrogel are suspect to different numbers of scatterers within the scattering volume due to different sample sizes (the microgel solution is loaded in a 1.5 mm thick capillary where the thickness of the hydrogel is ~5 mm).

Supplementary Note 2. The scattering patterns shown in Figure 3C of the main text and Supplementary Figure 8 arise due to the contrast between the hydrophobic micelle core and the O/water medium. We used cryo-SEM to confirm the nanostructure of the hydrogels, and

therefore, have validated modelling the scattering intensity profile by approximating the micelle size and the center-to-center micelle core distance as a Gaussian distribution, and that the micelles are noninteracting. Similar modelling has been utilized successfully in previously reported studies[5-7]. As a result, the total scattering intensity profile can be expressed as the product of hard sphere form factor, $P(q)$, (with a size distribution) and disordered spherical structure factor, $S(q)$, (with a distribution in the center-to-center micelle core distance) with a Percus–Yevick (PY) model[8].

The form factor, $P(q)$, is given by a modified version hard spheres form factor in which the spherical micelle core radius, R, is weighed with a Gaussian distribution, Supplementary Equation 4.

$$P(q, R_{\mathrm{c}}, \sigma_{\mathrm{c}}) = \frac{1}{\sqrt{2\pi}\sigma_{\mathrm{c}}} \sum_{R=R_{\mathrm{c}}-\sigma_{\mathrm{c}}}^{R_{\mathrm{c}}+\sigma_{\mathrm{c}}} e^{-\frac{1}{2}\left(\frac{R-R_{\mathrm{c}}}{\sigma_{\mathrm{c}}}\right)^2} \left(\frac{3(\sin(qR) - qR\cos(qR))}{(qR)^3}\right)^2 \tag{4}$$

In Supplementary Equation 4, R_{c} is the mean value of the hydrophobic micelle core radius R and σ_{c} is the standard deviation.

The disordered spherical structure factor, $S(q)$, shown in Supplementary Equation 5 is given by the Percus-Yevick (PY) model, where the inter-micelle correlation is represented by a hard-sphere potential that was first described by Ornstein and Zernike, and later approximated by Percus and Yevick with an analytical solution[8]. The model is also weighed with a Gaussian function, as the distribution in the micelle core sizes and the center-to-center distances of the micelles are assumed to be uncorrelated due to the kinetic mechanisms associated with hydrogel formation.

$$S(q, D_{\mathrm{hs}}, \varphi_{\mathrm{hs}}, \sigma_{\mathrm{hs}}) = \frac{1}{\sqrt{2\pi}\sigma_{\mathrm{hs}}} \sum_{D=D_{\mathrm{hs}}-\sigma_{\mathrm{hs}}}^{D_{\mathrm{hs}}+\sigma_{\mathrm{hs}}} \frac{e^{-\frac{1}{2}\left(\frac{D-D_{\mathrm{hs}}}{\sigma_{hs}}\right)^2}}{1 - Cs(q, D, \varphi_{\mathrm{hs}})} \tag{5}$$

Where Cs is defined in Supplementary Equation 6 as,

$$Cs(q, D, \varphi_{\mathrm{hs}}) = -24\varphi_{\mathrm{hs}} \left(\lambda_1 \left(\frac{\sin(qD) - qD\cos(qD)}{(qD)^3} \right) \right.$$

$$\left. -6\varphi_{\mathrm{hs}}\lambda_2 \left(\frac{(qD)^2\cos(qD) - 2qD\sin(qD) - 2\cos(qD) + 2}{(qD)^4} \right) \right.$$

$$-\frac{\varphi_{\text{hs}}\lambda_1}{2}\left(\frac{1}{(qD)^6}((qD)^4\cos(qD)-4(qD)^3\sin(qD)-12(qD)^2\cos(qD)\right.$$

$$\left.+24qD\sin(qD)+24\cos(qD)-24)\right)\quad(6)$$

and λ_1 (Supplementary Equation 7) and λ_2 (Supplementary Equation 8) are,

$$\lambda_1=\frac{(1+2\varphi_{\text{hs}})^2}{(1-\varphi_{\text{hs}})^4}\quad(7)$$

$$\lambda_2=-\frac{\left(1+\frac{\varphi_{\text{hs}}}{2}\right)^2}{(1-\varphi_{\text{hs}})^4}\quad(8)$$

In Supplementary Equation 5-8, D_{hs} is the mean value of the center-to-center distance D, σ_{hs} is standard deviation, and φ_{hs} is the hard sphere volume faction.

Finally, the total scattering intensity can be expressed as follows, Supplementary Equation 9,

$$I(R_{\text{c}},\sigma_{\text{c}},D_{\text{hs}},\varphi_{\text{hs}},\sigma_{\text{hs}})\approx P(q,R_{\text{c}},\sigma_{\text{c}})\cdot S(q,D_{\text{hs}},\varphi_{\text{hs}},\sigma_{\text{hs}})\quad(9)$$

The parameters used in the model were listed in Supplementary Table 7.

Supplementary Table 7. Parameters for modelling SAXS scattering patterns of the hydrogels.

**Sample**	R_{c}(nm)	σ_{c}(nm)	D_{hs}(nm)	σ_{hs}(nm)	φ_{hs}
IOI(17-53-17)	12	2	47.6	4.6	0.38
BOB(31-70-31)	27.5	2	86	0.5	0.36

The Young's modulus of the samples can be evaluated by fitting the initial part of the stress-strain curves using Supplementary Equation 10,

$$\sigma_{\text{eng}} = \frac{E}{3}\left(\lambda - \frac{1}{\lambda^2}\right) \tag{10}$$

Where σ_{eng} is engineering stress, λ is engineering strain, and E is Young's modulus.

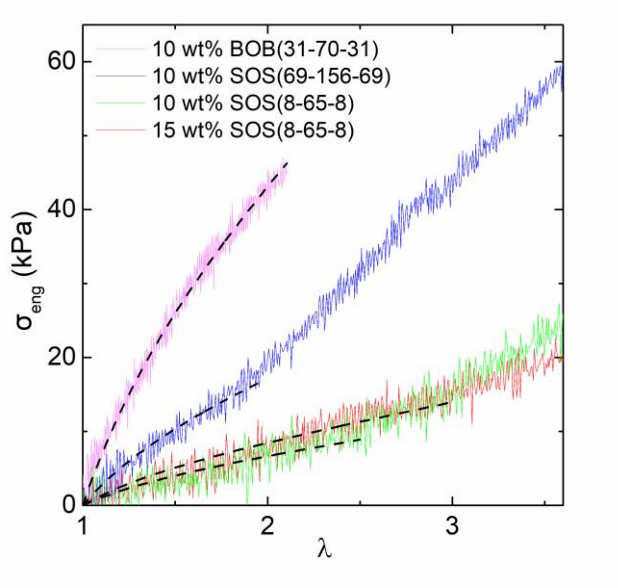

Supplementary Figure 9. Engineering stress versus elongation of hydrogels from using different triblock copolymer THF solutions. (λ = L/L_0, where L and L_0 are the length of the material at a specific stress and the original length, respectively). The low extension regime of the stress-strain curve was fitted using Supplementary Equation 10 to determine Young's modulus of the hydrogel samples. The modulus values are listed in Supplementary Table 8. The strain rate used was 3 min^{-1}.

Supplementary Table 8. Summary of the mechanical properties of a series of hydrogel samples. The values were calculated from strain-stress curves obtained at strain rate of 3 min^{-1}.

Sample	elongation at break	fracture energy (J m^{-2})	tensile strength (kPa)	Young's Modulus (kPa)
10 wt% BOB(31-70-31)	1.1	294	44.8	73.9
10 wt% SOS(69-156-69)	4.2	2016	100.8	29.4
10 wt% SOS(8-65-8)	5.2	1553	87.5	11.4
15 wt% SOS(8-65-8)	7.5	3066	118.1	14.5

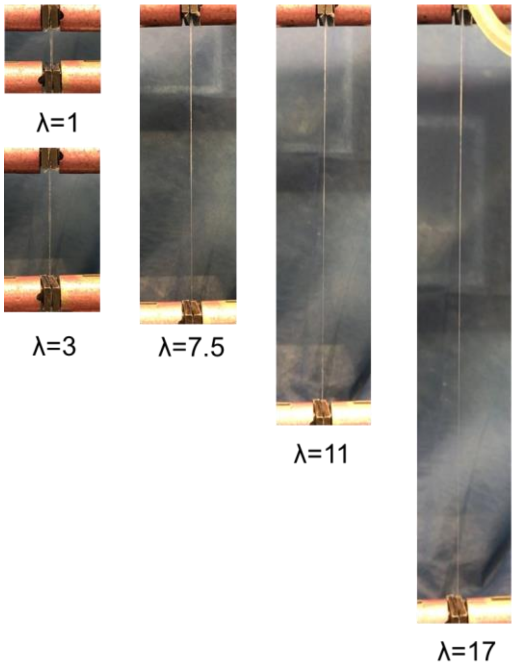

Supplementary Figure 10. Images of hydrogel fiber samples pulled (1 min^{-1}) at different elongation ratio values. The fibers were made from rapid injection of a 15 wt% SOS(8-65-8) THF solution.

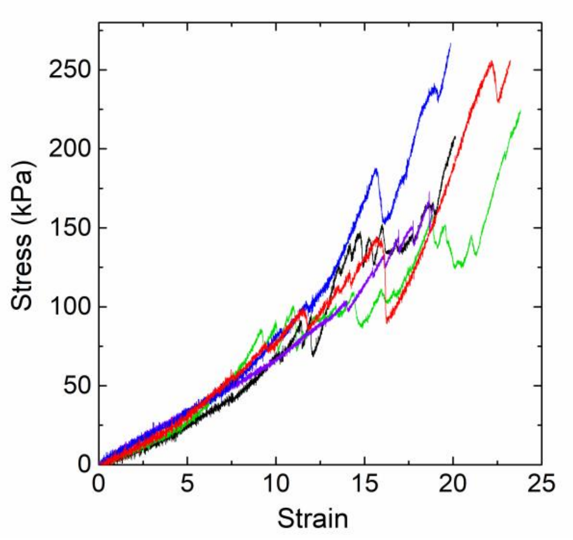

Supplementary Figure 11. Stress-strain curves of hydrogel fibers. The samples were fabricated using 15 wt% SOS(8-65-8) THF solution. The strain rate of the measurement was 1 min^{-1}.

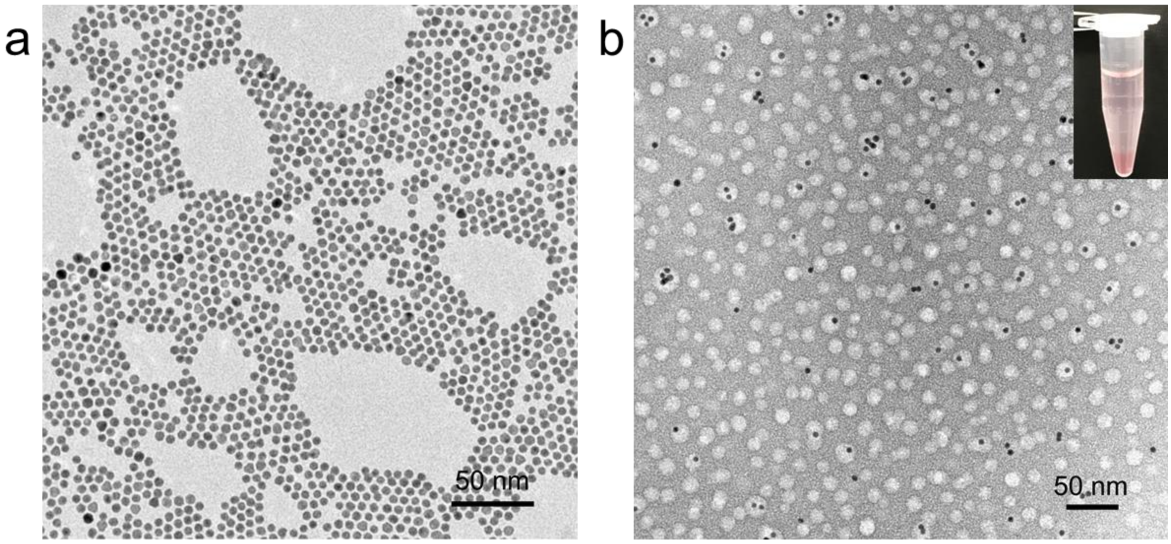

Supplementary Figure 12. Characterization of gold nanoparticles (AuNPs) for loading applications via rapid-injection. The gold nanoparticles (AuNPs) were synthesized according to previously reported procedures[9]. (a) TEM image of AuNPs dispersed in THF. The size of the AuNPs is 5.4 ± 0.5 nm. (b) Microgel from rapid-injection of a THF solution of 1.5 wt% SOS(8-65-8) with AuNPs (26.7 wt% with respect to polymer).

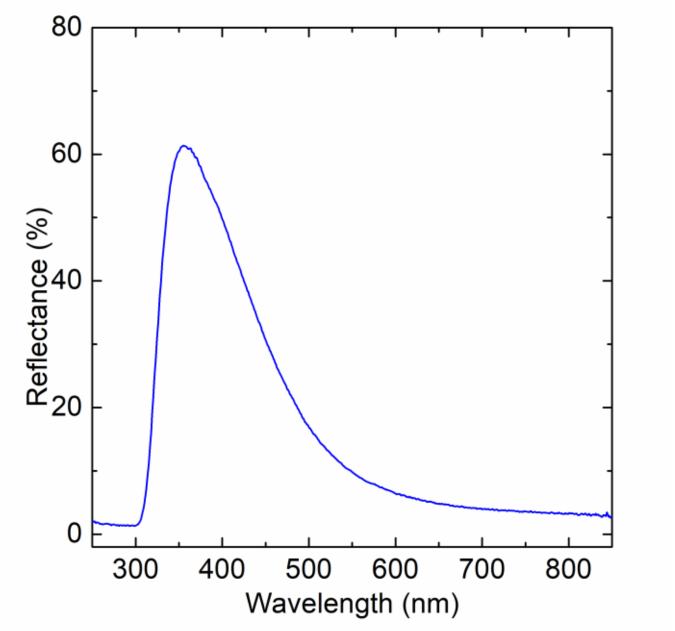

Supplementary Figure 13. UV-Vis reflection spectrum of the hydrogel exhibiting photonic band gap properties made from rapid injection of a THF solution of 10 wt% SOS(69-156-69). UV-Vis measurements (transmission and specular reflectance) were performed on a Perkin-Elmer Lambda 950 UV-Vis-NIR spectrophotometer. The hydrogel can reflect up to 60% of the light at wavelength of 350 nm (UVA region). The reflectance also suggests that at the visible spectrum, the hydrogel reflects both violet (380-450 nm) and blue (450-495 nm) light.

Supplementary References

1 Harding, S. *Refractive increment data-book for polymer and biomolecular scientists*. (Nottingham UK: Nottingham University Press, 2000).

2 Mark, J. E. *Physical properties of polymers handbook*. Vol. 1076 (Springer, 2007).

3 Hiemenz, P. C. & Lodge, T. P. *Polymer chemistry*. (CRC press, 2007).

4 Dormidontova, E. E. Role of competitive PEO− water and water− water hydrogen bonding in aqueous solution PEO behavior. *Macromolecules* **35**, 987-1001 (2002).

5 Wang, X., Dormidontova, E. E. & Lodge, T. P. The order− disorder transition and the disordered micelle regime for poly (ethylenepropylene-b-dimethylsiloxane) spheres. *Macromolecules* **35**, 9687-9697 (2002).

6 Taribagil, R. R., Hillmyer, M. A. & Lodge, T. P. Hydrogels from ABA and ABC triblock polymers. *Macromolecules* **43**, 5396-5404 (2010).

7 Guo, C. & Bailey, T. S. Highly distensible nanostructured elastic hydrogels from AB diblock and ABA triblock copolymer melt blends. *Soft Matter* **6**, 4807-4818 (2010).

8 Percus, J. K. & Yevick, G. J. Analysis of classical statistical mechanics by means of collective coordinates. *Phys. Rev.* **110**, 1-13 (1958).

9 Peng, S. *et al.* A facile synthesis of monodisperse Au nanoparticles and their catalysis of CO oxidation. *Nano Res.* **1**, 229-234 (2008).
