# Supplementary material for: Solvent-non-solvent rapid-injection for preparing nanostructured materials from micelles to hydrogels
Source: Nat Commun. 2019 Aug 26;10:3855. doi: 10.1038/s41467-019-11804-7 (PMC6710291; doi:10.1038/s41467-019-11804-7)
Supplement: Supplementary file 2 — Description of Additional Supplementary Files [file 41467_2019_11804_MOESM2_ESM.docx]

Description of Additional Supplementary Files

Supplementary Movie 1: Rapid-injection for making structural colored hydrogel fibers.

Supplementary Movie 2: Preparation of hydrogel printing that exhibits structural color.

Supplementary Movie 3: Complex mesh made from plasmonic and structural colored hydrogel fibers.

Supplementary Movie 4: Coating the substrate with a structural colored hydrogel layer.
